# hENT1 Testing in Pancreatic Ductal Adenocarcinoma: Are We Ready? A Multimodal Evaluation of hENT1 Status

**DOI:** 10.3390/cancers11111808

**Published:** 2019-11-18

**Authors:** Jerome Raffenne, Remy Nicolle, Francesco Puleo, Delphine Le Corre, Camille Boyez, Raphael Marechal, Jean François Emile, Peter Demetter, Armelle Bardier, Pierre Laurent-Puig, Louis de Mestier, Valerie Paradis, Anne Couvelard, Jean Luc VanLathem, John R. MacKey, Jean-Baptiste Bachet, Magali Svrcek, Jerome Cros

**Affiliations:** 1 Institut national de la santé et de la recherche médicale (INSERM) U1149, Inflammation research center, Beaujon’s Hospital, 92110 Clichy, France; raffenne.jerome@gmail.com (J.R.); camsboyer@gmail.com (C.B.); valerie.paradis@aphp.fr (V.P.); anne.couvelard@aphp.fr (A.C.); 2 Programme Cartes d’Identité des Tumeurs (CIT), Ligue Nationale Contre Le Cancer, 75013 Paris, France; Remy.Nicolle@ligue-cancer.net; 3 Gastroenterology Department, CHIREC Delta Hospital, 1160 Brussels, Belgium; francesco_puleo@hotmail.com (F.P.); ramarech@chu-tivoli.be (R.M.); 4 Sorbonne Paris Cité, Paris Descartes University, Georges Pompidou European Hospital, 75015 Paris, France; delphine.lecorre@yahoo.fr (D.L.C.); pierre.laurent-puig@parisdescartes.fr (P.L.-P.); 5 Department of Pathology, Ambroise Paré Hospital, 92100 Boulogne-Billancourt, France; jean-francois.emile@uvsq.fr; 6 Department of Pathology, Erasme Hospital, 1000 Brussels, Belgium; pieter.demetter@bordet.be; 7 Department of Pathology, Pitié-Salpetriére Hospital, 75013 Paris, France; armelle.bardier@aphp.fr; 8 Department of Gastroenterology, Beaujon Hospital–Paris University, 92110 Clichy, France; louis.demestier@aphp.fr; 9 Department of Pathology, Beaujon-Bichat Hospital–Paris University, 92110 Clichy, France; 10 Department of Gastroenterology and medical oncology, Erasme Hospital, 1000 Brussels, Belgium; JL.VanLaethem@erasme.ulb.ac.be; 11 Medical Oncoloy Cross Cancer Institute, Edmonton, AB T6G 1Z2, Canada; jrmackey@shaw.ca; 12 Department of Gastroenterology, Pitié-Salpetrière Hospital, Sorbonne Universités, UPMC Université, 75013 Paris, France; jean-baptiste.bachet@aphp.fr; 13 Dpt of Pathology, Saint Antoine Hospital, 75012 Paris, France; magali.svrcek@aphp.fr

**Keywords:** pancreatic adenocarcinoma, hENT1, Gemcitabine

## Abstract

Gemcitabine is still one of the standard chemotherapy regimens for pancreatic ductal adenocarcinoma (PDAC). Gemcitabine uptake into tumor cells is mainly through the human equilibrative nucleoside transport 1 (hENT1). It was therefore proposed as a potential predictive biomarker of gemcitabine efficacy but reports are conflicting, with an important heterogeneity in methods to assess hENT1 expression. A multicenter cohort of 471 patients with a resected PDAC was used to assess simultaneously the predictive value of the 2 best described hENT1 antibodies (10D7G2 and SP120). Three additional antibodies and the predictive value of hENT1 mRNA were also tested on 251 and 302 patients, respectively. hENT1 expression was assessed in 54 patients with matched primary tumors and metastases samples. The 10D7G2 clone was the only hENT1 antibody whose high expression was associated with a prolonged progression free survival and overall survival in patients who received adjuvant gemcitabine. hENT1 mRNA level was also predictive of gemcitabine benefit. hENT1 status was concordant in 83% of the cases with the best concordance in synchronous metastases. The 10D7G2 clone has the best predictive value of gemcitabine benefit in PDAC patients. Since it is not commercially available, hENT1 mRNA level could represent an alternative to assess hENT1 status.

## 1. Introduction

While gemcitabine was shown to be less effective than FOLFIRINOX in the adjuvant setting, it remains the preferred companion drug for nab-paclitaxel in advanced pancreatic ductal adenocarcinoma (PDAC), and in monotherapy, the standard for metastatic patients unfit for more aggressive chemotherapy regimens. Gemcitabine uptake into human cells is mainly due to the human equilibrative nucleoside transporter 1 (hENT1). It was therefore proposed as a predictive biomarker of gemcitabine benefit in resected and advanced PDAC. Recent meta-analyses confirmed this hypothesis [1,2]. In these studies, high hENT1 mRNA or protein levels were predictive of gemcitabine adjuvant benefit. Most of these results were obtained by using a non-commercially available murine monoclonal anti-hENT1 antibody (clone 10D7G2) [3,4,5,6]. The reported randomized phase III study conducted by Poplin et al. highlighted a new rabbit monoclonal hENT1 antibody (clone SP120), but hENT1 tumor expression was not found to predict gemcitabine benefit in patients with treatment-naive metastatic PDAC [7]. Similar results with the SP120 clone were reported by Ormanns et al. in advanced PDAC from the AIO-PK0104 phase III trial, by Sin et al. and our group on a small cohort of resected PDAC [6,8,9]. Recently, Kalloger et al. reported for the first time that in resected PDAC, the SP120 clone was predictive of a longer disease-free survival [10]. Because gemcitabine is still widely used in PDAC care, these results question the true predictive value of the commercially available rabbit SP120 monoclonal antibody and call for a definitive answer on how to best assess hENT1 status in PDAC. While these 2 hENT1 clones have been widely used, other groups suggested in small series that other hENT1 antibodies may be of interest [11,12]. In addition, some reports suggested that quantification of mRNA coding for hENT1 protein (*SLC29A1* gene) could be an alternate method [13,14].

Here, we report our experience with the 10D7G2 and SP120 antibodies on the largest multicenter series of resected PDAC (*n* = 471) together with the testing of three additional hENT1 commercial antibodies and mRNA levels. We also report for the first time the concordance of hENT1 expression in matched primary tumors and synchronous/metachronous metastases.

## 2. Results

### 2.1. Evaluation of the hENT1 SP120 Antibody Predictive Value

**Figure 1:**
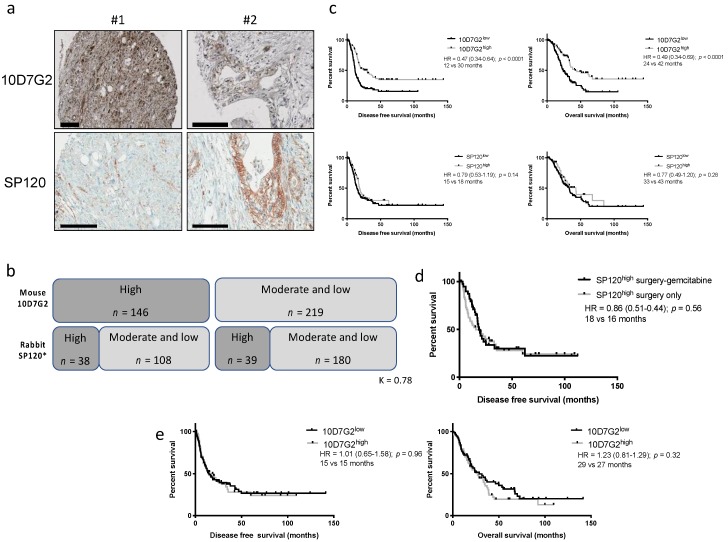
Comparison of the 10D7G2 and SP120 hENT1 clones. (**a**) Representative immunohistochemistry of 2 discordant cases between the 2 clones (black bar = 100 µm), (**b**) correlation between the 2 clones on the whole series, (**c**) disease free (left panels) and overall (right panels) survival in gemcitabine-treated patients. hENT1 high and low cases were defined with the 10D7G2 and the SP120 clones, (**d**) disease free and overall survival in patients not treated by gemcitabine. hENT1 high and low cases were defined with the 10D7G2 clone, (**e**) disease free (left panels) and overall (right panels) survival in adjuvant-free (only surgery) patients.

Patient characteristics for this cohort have already been reported and are summarized in Table S1. hENT1 status with the mouse 10D7G2 and the rabbit SP120 clones were assessed in 430 and 388 tumors, respectively. From a pure pathological point of view, the SP120 clone gave a signal that was more localized to the cell membrane compared to the 10D7G2, whose signal could also be diffused in the cytoplasm (Figure 1a). Both stainings were available for 365 tumors. Only 77 cases were fully concordant (38 10D7G2^high^/SP120^high^ and 39 10D7G2^low^/SP120^low^) using a 3-class scoring system (high/moderate low). When using a simpler 2-class scoring that combined low and moderate cases, 218 (59.7%) cases were concordant (Figure 1b). Interobserver reproducibility for the SP120 was good (K = 0.78). When only the patients who received a gemcitabine-based adjuvant treatment were considered (*n* = 259), high expression of hENT1 assessed by the 10D7G2 clone was a predictive biomarker of prolonged disease-free survival (DFS) (HR = 0.47 (95% CI, 0.34–0.64); *p* < 0.0001; 12 vs. 30 months) and overall survival (OS) (HR = 0.49 (95% CI, 0.34–0.69); *p* < 0.0001; 24 vs. 42 months) in univariate analysis (Figure 1c). In contrast, there was no predictive value of gemcitabine benefit with the rabbit SP120 clone on DFS (HR = 0.79 (95% CI, 0.53–1.19); *p* = 0.14; 15 vs. 18 months) and OS (HR = 0.77 (95% CI, 0.49–1.20); *p* = 0.28; 33 vs. 43 months). We also compared, like Kalloger et al., the patients displaying a SP120^high^ staining treated either by surgery-gemcitabine vs. surgery only but found no predictive value of gemcitabine benefit for this antibody (Figure 1d). Taken together, these results confirmed that the SP120 is not suitable for the assessment of the hENT1 status in resected PDAC in contrast to the mouse 10D7G2 clone. Of note the 10D7G2 clone had no prognostic value (DFS or OS) in the observed cohort (only surgery) confirming its pure predictive value (Figure 1e).

### 2.2. Evaluation of Additional hENT1 Antibodies Predictive Value

**Figure 2:**
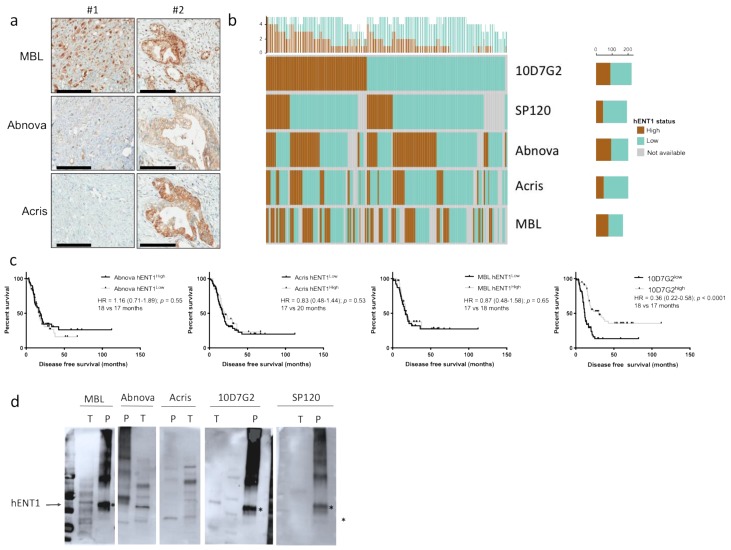
Comparison of all hENT1 clones. (**a**) Representative immunohistochemistry of the 2 cases showed in Figure 3 with the 3 additional hENT1 clones (black bar = 100 µm), (**b**) correlation between the 5 hENT1 clones. Each vertical line represents one tumor with its hENT1 status across all five antibodies (high = brown color, low = green color, grey = indeterminate). The top panel summarizes each tumor with the number of positive and negative antibodies, (**c**) disease free survival in gemcitabine-treated patients. hENT1 high and low cases were defined with the 10D7G2 and the 3 additional clones, (**d**) Western blot with the 5 clones on tumor protein extracts (T) and on purified hENT1 protein (P). See complete membranes in Figure S2.

We then evaluated 3 additional commercial antibodies in the patients from the 2 largest centers of the cohort (*n* = 251). The polyclonal antibodies from MBL™ and Abnova™ gave a more diffuse cytoplasmic and membranar signal than the polyclonal antibody from Acris™ (Figure 2a). Similar to the SP120, the concordance with the mouse 10D7G2 was poor (Figure 2b). In gemcitabine-treated patients (*n* = 127), none of the antibodies had a predictive value of gemcitabine benefit (DFS) in contrast to the 10D7G2 (Figure 2c). To better address the specificity of all these antibodies, we performed a Western blot using a commercially available purified hENT1 extract and a tumor extract from a 10D7G2^high^/SP120^high^ case. All clones recognized the expected 50 kD band corresponding to hENT1 in the purified extract lane (Figure 2d). hENT1 is a glycosylated protein and other bands (higher molecular weight) were detected in the purified extract by all antibodies, albeit with different affinity depending on the clones. In the PDAC protein extract, the 10D7G2 was the most specific antibody detecting only the expected 50kD band while the other clones, except the SP120, detected multiple bands that did not correspond to the probably glycosylated forms of hENT1, questioning their specificity.

### 2.3. Evaluation of hENT1 mRNA Predictive Value

**Figure 3:**
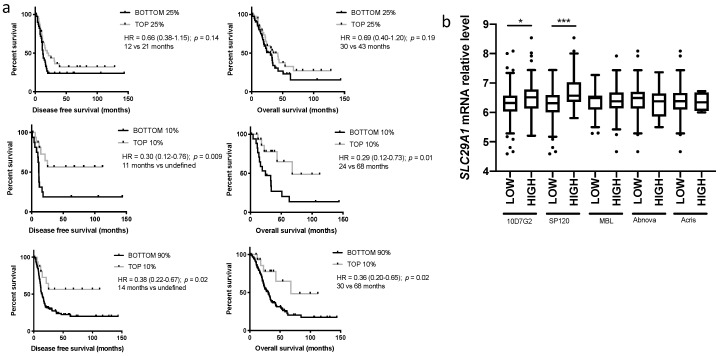
Predictive value of hENT1 mRNA level and correlation with immunohistochemistry. (**a**) Disease free survival in gemcitabine-treated patients according to the hENT1 status defined by the hENT1 (*SLC29A1*) mRNA level top 25% vs. bottom 25% (top panel), top 10% vs. bottom 10% (middle panel), top 10% vs. the rest of the cohort (bottom panel), (**b**) level of *SLC29A1* mRNA level in hENT1 high and low cases defined by immunohistochemistry with the different clones. * *p* < 0.05, *** *p* < 0.0001.

Whole tumor mRNA levels from microarray data were available for 164 patients that were treated with an adjuvant gemcitabine-based treatment. There was no difference in disease free survival or overall survival using the median *SLC29A1* mRNA value as a threshold for hENT1 high and low tumor. In contrast, if a more stringent threshold was used (top 25% vs. bottom 25% *SLC29A1* expression levels), there was a trend toward a predictive value of high levels of *SLC29A1* mRNA (Figure 3a). Further increase of the threshold (top 10% vs. bottom 10% or bottom 90% *SLC29A1* mRNA expression levels) allowed us to select a population of exceptional responders to gemcitabine (undefined median disease-free survival and median overall survival superior to 5 years) (Figure 3a). For a subset of patients (90 patients from center 4), a second mRNA extraction was performed and the *SLC29A1* mRNA level assessed by a more conventional Taqman-based RT-qPCR. There was a good correlation with both techniques (*r* = 0.42, *p* < 0.001) suggesting that this might represent an alternative method. *SLC29A1* mRNA levels were higher in hENT1^high^ cases compared to hENT1^low^ defined by IHC stainings with both the mouse and the rabbit clones. Of note, the mRNA level difference between hENT1^high^ and hENT1^low^ cases was more important with the SP120 than the 10D7G2 clone. With the three other clones, there was no difference in mRNA levels between hENT1^high^ and hENT1^low^ tumors.

### 2.4. Evaluation of hENT1 Expression in Metastasis and Correlation with the Primary Tumor

Out of the 66 patients selected, only 54 had available samples of their primary tumor and metastases in which the hENT1 status could be determined by IHC (mouse clone 10D7G2) (Table S2). Metastases were synchronous in 32 cases (59%). For two patients, two and three metastatic sites respectively were available for assessment. All the metastatic samples from one patient had similar hENT1 status. Concordance of hENT1 status between primary tumors and metastases was excellent (45/54; 83% overall) with a slightly higher concordance for synchronous metastases (30/32; 94% vs. 15/22; 68%) (Table 1). Regarding the discordant metachronous metastases, 5/7 (71%) lost their hENT1 expression compared to the primary tumor while the two remaining cases had a higher expression in the metastases compared to the primary tumor. Assessment of the hENT1 status on surgical specimens or FNA had no impact on the concordance between localizations.

## 3. Discussion

While effective new chemotherapy regimens have been proposed in metastatic PDAC, gemcitabine remains the backbone of all combinations with nab-paclitaxel and is still the main adjuvant treatment as well as a treatment of choice for patients unfit for more aggressive chemotherapies. In patients with metastatic disease, 5-FU was shown to be almost as effective as gemcitabine in combination with nab-paclitaxel [15]. It is therefore of the utmost importance to develop robust predictive biomarkers to select the best candidates for either therapy. hENT1, encoded by the *SLC29A1* gene is the main transporter of gemcitabine within the tumor cells and was therefore proposed as a good biomarker candidate. Yet there is no consensus on how to best assess hENT1 expression and reports of hENT1 predictive value have been conflicting. All studies, including this study, suggested that hENT1 status assessed with the mouse 10D7G2 clone was predictive of gemcitabine benefit [3,4,5]. Unfortunately, this clone is not commercially available precluding its use. This led to the test of other commercial clones. Amongst them, the rabbit SP120 has been the most studied but with inconsistent results. While Sinn et al. and Ormanns et al. found that this clone had no predictive value of gemcitabine benefit, Kalloger et al. recently reported the opposite on a small cohort of 54 gemcitabine treated patients [6,8,10].

This study provides the largest assessment of both antibodies in a multicentric cohort of 472 patients. As reported before, the high expression of hENT1 assessed with the mouse 10D7G2 was predictive of a prolonged DFS and OS when, and only when, treated with gemcitabine. In contrast, we found results with the rabbit SP120 in concordance with those of Sinn et al. and Ormanns et al., namely an absence of predictive value regardless of the method (comparison of hENT1 high and low in the gemcitabine-treated group or comparison of hENT1 high in the gemcitabine-treated group vs. observed group). We found, like Kalloger et al., that the number of hENT1 high tumors defined by the SP120 clone was much lower than with the 10D7G2. If it was a sensitivity problem of the SP120 clone, we would expect only the tumors with the highest expression to be positive. This would have led to the detection of a reduced group but with hENT1-“very high” expression and an expected outstanding response to gemcitabine. One hypothesis is that these two clones recognize different epitopes. We did not perform dual immunofluorescence to assess whether the subcellular localizations of the recognized proteins were identical but since serial sections of TMAs were used (only 5 μm apart), it is unlikely that a concordant case would have been missed because of spatial heterogeneity in the staining. While those two antibodies seem to have a similar recognition profile on a Western blot with purified hENT1 extract, the SP120 stained a band in purified tumors of a higher molecular weight. The peptide sequence used to generate the SP120 antibody is unavailable in contrast to that of the 10D7G2. hENT1 is a highly glycosylated protein and the SP120 may recognize a particular form of hENT1 that is not fully active. We found similar results with the other clones that we tested. All of them detected a smaller proportion of hENT1 high tumors and this was not due to a lack of sensitivity nor to a lack of immunoreactivity, as we obtained strong staining with all clones.

hENT1 proper assessment may pave the road to better selection of patients but there are other important players in gemcitabine sensitivity/resistance. In tumor cells, for instance, gemcitabine-metabolizing enzyme levels and activity such as CDA, dCK or Nt5c1A/Nt5c3 may explain the discrepancy between high hENT1 level and poor response to gemcitabine [3,16]. In addition, it should be noted that cells from the microenvironment also participate in gemcitabine resistance such as cancer associated fibroblasts (CAF) and macrophages [17]. For instance, Hessmann et al. reported that CAF are able to take up and inactivate gemcitabine, reducing the available amount for tumor cells [18]. One limitation of this work is that we did not assess hENT1 level in cells from the microenvironment but the aim of this work was to assess if a simple, single population-hENT1 scoring could predict gemcitabine efficacy in a routine setting. Multiple population scorings such as that of PDL1 are complicated and often lack reproducibility. hENT1 whole tumor status determination through mRNA quantification could be an alternative to immunohistochemistry. We showed here in the largest evaluated cohort of gemcitabine-treated patients (*n* = 164), an SLC29A1 mRNA level-dependent response to gemcitabine. It should be noted that this study was performed on routine formalin-fixed paraffin-embedded (FFPE) samples and with only macrodissection, like it is classically performed in pathology departments. The threshold necessary to achieve statistical significance was higher than with immunohistochemistry (top 25% for OS and top 10% for PFS) but no microdissection was performed here, leading to heavy microenvironment contamination and a possible falsely decreased normalized SLC29A1 mRNA level. There was a good correlation between the SLC29A1 mRNA level determined by microarray and RT-qPCR. Both techniques were performed on FFPE material suggesting that SLC29A1 whole tumor mRNA level could be determined in a routine setting with a minimal macrodissection to avoid normal tissue contamination. Alternatives would be to use RNA-based gemcitabine sensitivity signatures or RNA hybridization techniques. While the former seems easily achievable once the technique has entered routine pathology laboratories, the robustness of the former (developed on tumor organoids) needs to be evaluated, especially on FFPE samples with heavy contamination from the microenvironment [19]. The SLC29A1 mRNA levels were correlated to the antibodies staining but surprisingly the concordance appeared better with the rabbit SP120. This may suggest that the SP120 recognized an unprocessed form of hENT1 directly linked to mRNA level while the 10D7G2, a stabilized form, was possibly more active to explain its better predictive value. Again, because mRNA was extracted from the whole tumor, it may explain in part the low correlation between mRNA and protein levels.

Finally, we provide here the first and largest assessment of hENT1 status on coupled samples from primary and metastatic localizations. These data showed that when facing synchronous metastases, the choice of the localization and the technique (fine needle biopsy vs. surgical specimen) for hENT1 status determination is not important, the concordance being excellent. On the contrary, in metachronous metastases, there was a discordance in 30% of the cases prompting for a preferred determination on the metastatic material even if a new sampling is required, especially since metastases tended to lose hENT1 expression compared to their primary counterpart. Another possibility that we could not validate because of lacking clinical data is that adjuvant gemcitabine-based treatment may have selected hENT1^low^ clones in the metachronous metastases. In addition, cells from the microenvironment also participate in gemcitabine resistance through drug uptake and inactivation [18].

## 4. Material and Methods

### 4.1. Patients

Our institutional review boards (Comité de Protection des Personnes, Ile de France IV) approved this translational study (Ref 2010/01 NICB and 2014/59NICB). The study included 471 consecutive and unselected patients who underwent, between September 1996 and August 2009, curative intent surgery for PDAC at 5 university centers with expertise in management of PDAC and gave their informed consent. The characteristics of this cohort were already reported and are presented in Table S1 [3]. Patients did not receive any preoperative chemotherapy or chemoradiotherapy. The flow chart of the study is presented in Figure S1. In addition, 66 patients with a matched sample available of their primary tumor and at least one metastasis (either synchronous or metachronous) were selected (Table S2).

### 4.2. Immunohistochemistry

We used the same tissue microarrays from our previously published series [3]. Briefly, 3–5 cores/tumor (1mm in diameter) were made in the FFPE tumor blocks and assembled in 17 TMAs. The staining with the murine anti-hENT1 antibody was performed by Mackey JR and colleagues in their laboratory as previously described [3]. The immunohistochemistry with all the other anti-human hENT1 antibodies were performed on Ventana immunohistochemistry automates (Benchmark GX, Tucson, AZ, USA). Antibody dilution, antigen retrieval and detection systems are reported in Table S3. The intensity of hENT1 staining was ranked from 0 to 2, as previously described [9]. A case was scored “low” if more than 95% of tumor cells had a staining intensity of 0, “high” if >50% of tumor cells had a staining intensity of 2, and all other patterns were scored “moderate”. Each TMA core was not scored separately. We combined all the cores from each tumor to compute the percentage of positive cells and their intensity. For the primary tumor/metastases study, we analyzed whole slides and computed the average percentage of positive cells and their intensity. Antibodies staining was assessed independently by 2 pathologists with long-standing experience in pancreatic pathology (J.C. and M.S.), and blinded to the patient outcomes. Diagnostic reproducibility was assessed using Fleiss k. Discordant cases were reviewed and a consensus was reached. The hENT1 staining results were correlated with DFS and OS following the surgery. hENT1 high cases (score 2) were compared to pooled moderate and low cases. Cox proportional hazards regression model was used for the analysis of survival and for estimating hazard ratios (HR) with 95% confidence intervals (CI).

### 4.3. Western Blot Analyses

Total proteins were extracted in 2% Tris HCL 2M pH 7.5, 2% EDTA 0.5 M pH 8, 0.5% EGTA, 1% Triton X100, 0.5% protease inhibitor and 0.5% phosphatase inhibitor (Sigma-Aldrich, Saint Louis, MO, USA). A total of 10 μg of proteins were separated in SDS-PAGE 4–15%, transferred to PVDF membrane and saturated overnight at 4 °C in TBST-BSA5% (Tris buffer saline plus 0.05% Tween 20 and 5% bovine serum albumin). Membranes were incubated with the primary antibody for 2 h at room temperature at the dilution described in Table S3 and revealed with the corresponding human recombinant peroxidase-linked secondary antibody. Acquisitions were carried out using electro-chemi-luminescence Clarity™ ECL Western Blotting Substrate (Biorad™, Marnes-la-coquette, France) with a ChemiDoc touch imaging system (Biorad™). All experiments were performed in triplicate.

### 4.4. mRNA Analysis

Formalin-fixed paraffin-embedded (FFPE) blocks from 232 resected PDAC from the French cohort (Pitié Salpètrière, Saint Antoine, and Ambroise Paré hospitals) and 80 from the Brussels cohort were retrieved for combined DNA/RNA extraction. A representative tumor block was selected by specialist pancreatic pathologists (P.D., L.V., M.S., J.C., M.G.G.) after examination of hematoxylin and eosin (H & E) stained slides. In the Brussels cohort, slides were manually microdissected to enrich for neoplastic cells. In the French cohort, 2 cores (diameter of 1.5 mm) were extracted from the selected paraffin block in a tumor-rich area. DNA/RNA was extracted using the ALLPrep FFPE tissue kit (Qiagen™, Venlo, The Netherlands) following manufacturer’s instructions. For the whole cohort, a total of 100 ng of RNA was used as starting material for biotin-labelled cDNA preparation. For the labelling procedure, the Affymetrix Sensation FFPE and 3′IVT kit (Affymetrix™, Santa Clara, CA, USA) was used according to the manufacturer’s instructions. Transcriptomic profiles were acquired using Affymetrix HG-U219 microarrays (*n* = 312), normalized using RMA and batch effects corrected after removing 3 low quality profiles. For the Saint Antoine cohort, RNA was extracted from a 1.5 mm core taken in the same area with the Qiagen™ RNA FFPE minikit. Reverse transcription was performed with random primers and the superscript III (Life technologies™, Carlsbad, CA, USA). Relative mRNA levels were measured by qPCR in triplicate using gene-specific probe sets (SLC29A1 hs01085704_g1and GAPDH 02758991_g1) and the TaqMan Universal Master Mix II with UNG (Applied Biosystems™, Foster City, CA, USA) on a LightCycler 96 detection system (Roche Diagnostic™, Meylan, France).

## 5. Conclusions

In conclusion, we provided here the most comprehensive multimodal analysis of hENT1 expression in PDAC, and did so in the largest cohort to date. Our data showed that the mouse 10D7G2 antibody is the only one with a robust predictive value of gemcitabine benefit. Since this clone is not available commercially, we proposed that *SLC29A1* mRNA level determination could be an alternative.

## Figures and Tables

**Table 1:** hENT1 status in paired primary tumor and metastases.

Type of Couples	Concordance Between Primary Tumor and Metastases (0–1 vs. 2)
All couples	45/54: 83%
Synchronous (*n* = 32)	30/32: 94%
FNA (*n* = 17)	16/17: 94%
Autopsy (*n* = 5)	4/5: 80%
Metachronous (*n* = 22)	15/22: 68%

## Supplementary Materials

The following are available online at https://www.mdpi.com/2072-6694/11/11/1808/s1, Figure S1: Flow chart of the study, Figure S2: Raw complete membranes of the Western blot presented in Figure 2d, Table S1: Patient characteristics in the whole cohort and according to the treatment, Table S2: Sample description of the paired primary tumor-metastase(s) cohort, Table S3: Antibodies used in the study.
