# Supplementary material for: hENT1 Testing in Pancreatic Ductal Adenocarcinoma: Are We Ready? A Multimodal Evaluation of hENT1 Status
Source: Cancers (Basel). 2019 Nov 18;11(11):1808. doi: 10.3390/cancers11111808 (PMC6896053; doi:10.3390/cancers11111808)
Supplement: Supplementary file 1 [file cancers-11-01808-s001.pdf]

Supplementary Materials: hENT1 Testing in Pancreatic Ductal Adenocarcinoma: Are We Ready? A Multimodal Evaluation of hENT1 Status

Jerome Raffenne, Remy Nicolle, Francesco Puleo, Delphine Le Corre, Camille Boyez, Raphael Marechal, Jean François Emile, Peter Demetter, Armelle Bardier, Pierre Laurent-Puig, Louis de Mestier, Valerie Paradis, Anne Couvelard, Jean Luc VanLathem, John R. MacKey, Jean-Baptiste Bachet, Magali Svrcek and Jerome Cros

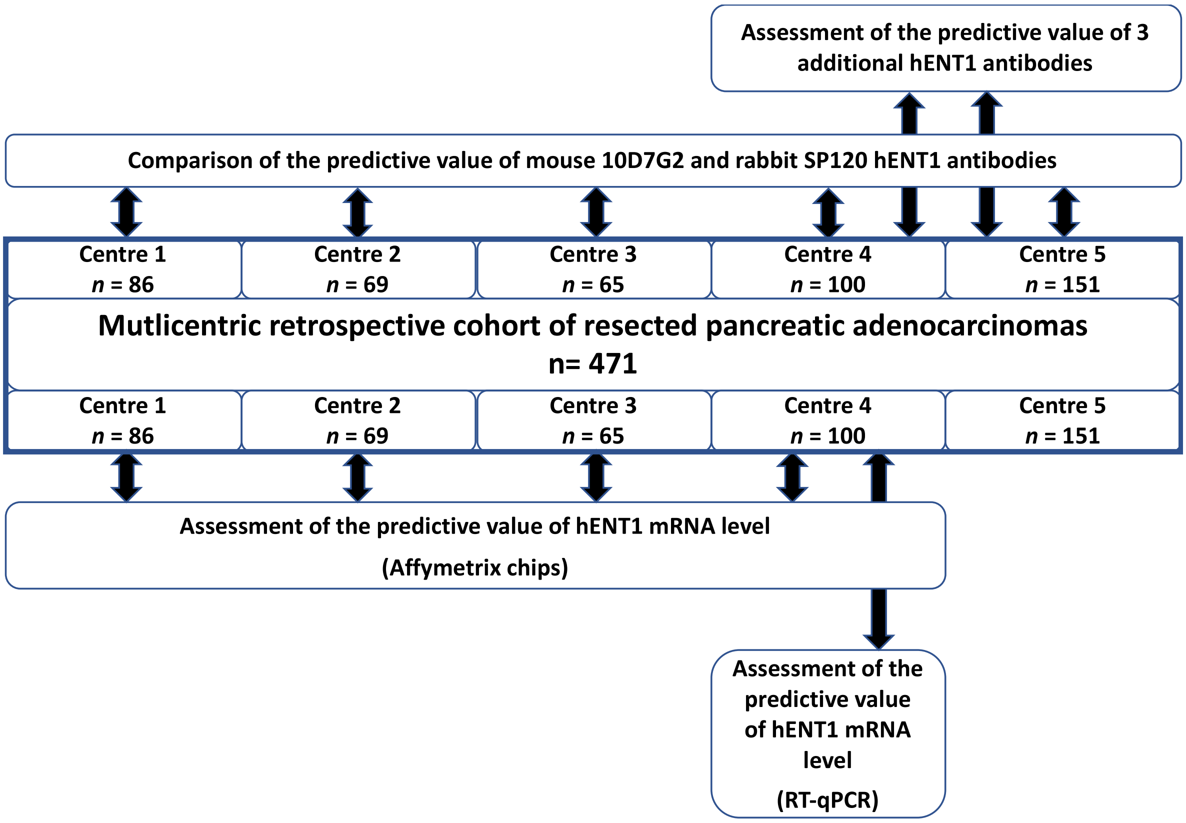

Figure S1. Flow chart of the study.

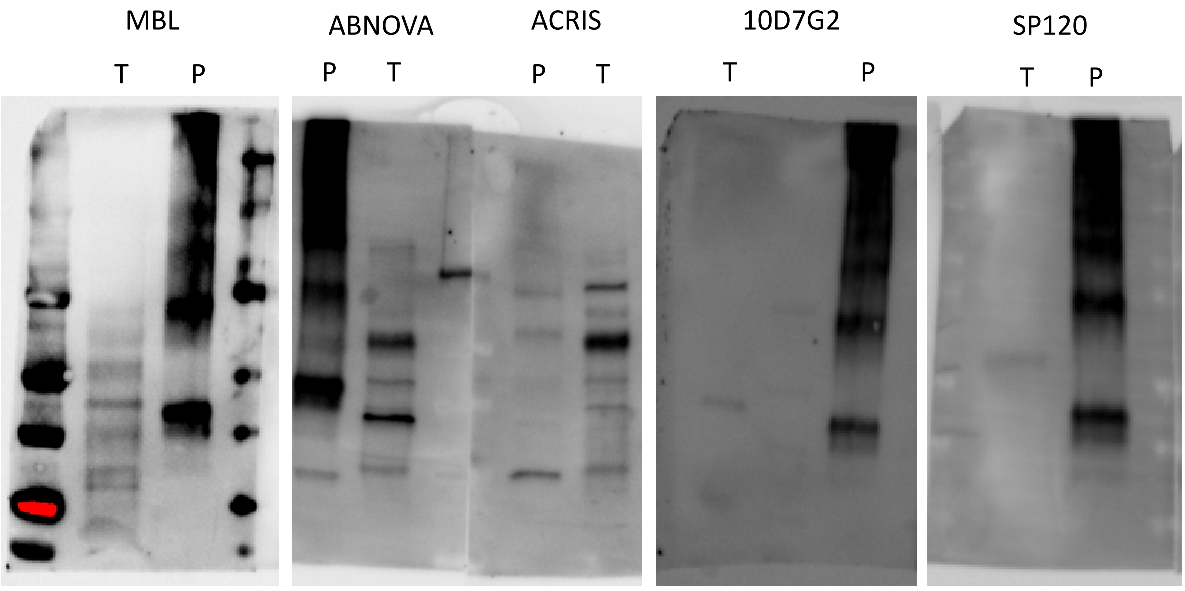

Figure S2. Raw complete membranes of the Western blot presented in Figure 2d.

Table S1. Patient characteristics in the whole cohort and according to the treatment.

**Variables**	**No Gemcitabine-Based Therapy (n = 191)**	**Gemcitabine-Based Therapy (n = 243)**	**All (n = 434)**	**p value χ^2 test**
Center				
1	29 (15.2)	39 (16.1)	68 (15.7)	0.327
2	33 (17.3)	35 (14.4)	68 (15.7)	
3	69 (36.1)	78 (32.1)	147 (33.9)	
4	20 (10.5)	42 (17.3)	62 (14.3)	
5	40 (20.9)	49 (20.2)	89 (20.5)	
Sex				
Female	84 (44)	114 (46.9)	198 (45.6)	0.542
Male	107 (56)	129 (53.1)	236 (54.4)	
Race				
White	185 (96.8)	234 (96.3)	419 (96.5)	0.95
Black	4 (2.1)	6 (2.6)	10 (2.3)	
Other	2 (1.1)	3 (1.1)	5 (1.2)	
Surgery				
Duodenopancreatectomy	165 (86.4)	213 (87.7)	378 (87.1)	0.06
Distal pancreatectomy	24 (12.6)	20 (8.2)	44 (10.1)	
Total pancreatectomy	2 (1.1)	10 (4.1)	12 (2.8)	
Resection margins				
R0	160 (85.1)	188 (77.7)	348 (80.9)	0.052
R1	28 (14.9)	54 (22.3)	82 (19.1)	
Tumor differentiation				
Well differentiated	88 (48.1)	99 (41.8)	187 (44.5)	0.433
Moderately differentiated	72 (39.3)	104 (43.9)	176 (41.9)	
Poorly differentiated	23 (12.6)	34 (14.4)	57 (13.6)	
Tumor stage				
T1/T2	37 (19.4)	39 (16.1)	76 (17.5)	0.366
T3	154 (80.6)	204 (84)	358 (82.5)	
Lymph nodes Negativ				
Negative, N0	60 (31.4)	56 (23.1)	116 (26.7)	0.051
Positive, N1	131 (68.6)	187 (77)	318 (73.3)	
Tumor size (mm)				
≤30	116 (64.1)	126 (56)	242 (59.6)	0.099

>30	65 (35.9)	99 (44)	164 (40.4)
Median (minimum/maximum)	30 (7/100)	30 (10/150)	30 (7/150)
Mean (SD)	31.2 (15)	33.5 (16.2)	32.5 (15.7)
Treatment regimens			
Gem monotherapy 6 mo		103 (42.4)	
Gem 4 mo followed by RT + 5-FU		11 (4.5)	
Gem 3 mo followed by RT + 5-FUc		80 (32.9)	
Gem 2 mo followed by RT + gem		14 (5.8)	
Gem and oxaliplatin 3 mo		35 (14.4)	

All values are expressed as *n* (%) unless otherwise noted. RT, radiation therapy; 5-FU, 5-fluorouracil; Gem: gemcitabine

Table S2. Sample description of the paired primary tumor-metastase(s) cohort.

**Paired Primary Tumor-Metastase(s)**	***n* = 54**
Primary tumor obtained from	
pancreatectomy (surgery)	29 (54%)
FNA	20 (37%)
autopsy	5 (9%)
Metastases obtained from	
surgical resection	9 (17%)
FNA/biopsy	40 (74%)
autopsy	5 (9%)
Synchronous	32 (59%)
Metachonous	22 (41%)

Table S3. Antibodies used in the study.

**Antibodies**	**Clone/Ref**		**Species**	**Suppliers**	**Dilution Factor**	
					**IHC**	**WB**
hENT1	10D7G2	-	murine	Gifte of Mackey JR	1/2	1/200
	SP120	M4200	rabbit	Spring Bioscience	1/50	1/1000
	-	PAB2255	rabbit	Abnova	1/100	1/500
	-	MC-9777	rabbit	MBL	1/500	1/500
	F12	Fsc-377283	murine	Santa Cruz	1/100	1/1000
	-	11337-1-AP	murine	Acris	1/50	1/1000
HRP Anti-Mouse IgG	-	ab131368	rat	Abcam	-	1/2000
HRP Anti-rabit IgG	-	7074S	goat	Cell signaling	-	1/2000

© 2019 by the authors. Licensee MDPI, Basel, Switzerland. This article is an open access article distributed under the terms and conditions of the Creative Commons Attribution (CC BY) license (http://creativecommons.org/licenses/by/4.0/).
